# Barriers of persistent long-lasting insecticidal nets utilization in villages around Lake Tana, Northwest Ethiopia: a qualitative study

**DOI:** 10.1186/s12889-019-7692-2

**Published:** 2019-10-16

**Authors:** Asmamaw Malede, Mulugeta Aemero, Sirak Robele Gari, Helmut Kloos, Kassahun Alemu

**Affiliations:** 1 Ethiopian Institute of Water Resources, Addis Ababa University, Addis Ababa, Ethiopia; 2 Department of Medical Parasitology, School of Biomedical & Laboratory Sciences, University of Gondar, Gondar, Ethiopia; 3 Department of Epidemiology and Biostatistics, University of California, San Francisco, USA; 4 Department of Epidemiology and Biostatistics, Institute of Public Health, University of Gondar, Gondar, Ethiopia

**Keywords:** Malaria, Mosquitoes, LLINs, In-depth interviews, Conical nets, Bedbug infestation

## Abstract

**Background:**

Malaria remains a major public health problem in Ethiopia. The use of LLINs is an effective approach to reducing transmission. Persistent use of LLINs is determined by numerous factors. Quantitative studies have assessed LLIN ownership and utilization, but the behavioral, socio-cultural, socioeconomic and net distribution contexts that impact their use have not been examined in depth. This study aimed to explore barriers of persistent LLIN use among communities around Lake Tana.

**Methods:**

Twenty-three community residents who owned LLINs (15) or not (8) during the study period and 38 key informants were interviewed from April to June 2017. Phenomenological study was employed to explore the local contexts and factors that influence persistent use of LLINs. Individuals were purposefully selected to capture different views. Community residents were selected based on their permanent residence and LLIN use experience. Key informants were health extension workers, local leaders, students, and health professionals. The data were managed using QSR International NVivo Version 10 software and coded, and themes were identified.

**Results:**

Killing ability of nets against arthropods other than mosquitoes reportedly made use of LLINs a favored malaria prevention method despite their ineffectiveness after 3 months. Conical nets were preferred due to their compatibility with varied sleeping structures. Numerous factors influenced persistent use, notably erroneous perceptions about LLINs, malaria and mosquitoes; bedbug infestation; inconvenience; unintended uses; distribution problem of nets; and socio-cultural and economic factors. Unintended uses were often associated with local needs and seldom linked with social issues and deficiencies in information about malaria and LLINs. Collateral benefits were considered important, principally in terms of disinfestation of bedbugs.

**Conclusions:**

Non-persistent LLIN use was associated with inconvenient bed net design and early damage; non-potency of the insecticide against other arthropods; facilitation of bedbug infestation; unintended uses; wrong perceptions about malaria, mosquitoes, and LLINs; and inadequate follow-up regarding LLINs utilization. Distribution of conical nets and provision of adequate information on LLINs and malaria may promote persistent use. Using an insecticide that also kills arthropods other than mosquitoes may reduce unintended uses and increase persistent use.

## Background

Malaria is a major public health concern in Ethiopia [1]. The use of long-lasting insecticidal nets (LLINs) is a widely accepted [2] and commonly available intervention for preventing malaria in Africa [3]. It is a primary prevention and control strategy in Ethiopia [1, 4] and promoted as an effective approach to minimizing risk of malaria transmission. Worldwide, studies have revealed major benefits from LLINs ownership and use [5].

According to the 2015 National Malaria Indicator Survey, only 64% of Ethiopian households (HHs) owned at least one LLIN and only 40% of the population slept under LLINs the night before the survey, below the goals of 100% ownership and 80% utilization. The proportion of HHs having at least one LLIN for every two people was 31.7% nationally and 38% in Amhara Region in 2015. In Amhara Region, where this study was carried out, 72.9% of HHs owned one LLIN and 43.4% of the population slept under the LLINs the night before the interview. The use of existing LLINs in Amhara Region was 56%, the lowest nation-wide [1]. Evidence from rural Ethiopia showed variable LLIN coverage and utilization [6]. A recent study reported that only 12% of HHs owned one or more LLINs [7].

Persistent utilization of LLINs is determined by an array of factors, including, first and foremost, insufficient access to LLIN within HHs and mal-distribution of LLINs, and availability or ownership of LLINs [1, 4, 8–10], followed by seasonality and overall mosquito abundance. Misconceptions about malaria risk, symptoms, and transmission; community norms and values; and gender or age priorities contribute to nonuse of LLINs. In Ethiopia, the groups most vulnerable to malaria and therefore prioritized for LLIN usage are children under 5 years of age and pregnant women. The age or physical condition of the nets, perceptions of net ineffectiveness, structural incompatibilities and practical issues, and use of nets for unintended purposes affect utilization [1, 4, 11–18]. The most frequently cited reasons for non-persistent use of nets are perceived low mosquito abundance and stuffiness, usually during hot weather [11]. Promotion of net use in Ethiopia and other countries has focused almost exclusively on malaria prevention. Therefore, use of nets may be considered unimportant in communities in which malaria is no longer seen as a serious health problem. Moreover, LLINs have a short lifespan, developing tears and holes and losing insecticide over time; field studies show that the useful life of a net may vary between 18 months and 7 years, with a mean expected life span of 3 years [19, 20]. However, a cohort study in south-central Ethiopia revealed only 4% of 1532 distributed LLINs met the criteria for functional survival by the 24th month, with a median functional survival time of 12 months [21].

Parallel matched case-control studies in villages around Lake Tana identified risk factors for malaria in a meso-endemic setting. The results showed that under-five age (36–59 months), travel to malarious lowlands, and inadequate malaria information were independent predictors. Ownership and use of LLINs were low; 69.5% of the participants had no nets [22, 23]. These studies provide information on determinants of malaria prevalence, but they did not explore the behavioral, sociocultural, and socioeconomic contexts explaining why communities did not use LLINs persistently. Thus, factors that influence persistent LLIN utilization in specific areas need to be investigated to facilitate malaria elimination. The aim of the present study was to explore context-based barriers to persistent LLINs use among residents in villages around Lake Tana. Findings of this study may have implications for malaria elimination strategies.

## Methods

### Study setting and population

The study was carried out in seven *kebeles* (the lowest administrative units in Ethiopia), including Lemba-Arebaytu, Layeye-Dugie, Zengage, Jeja-Bahriegeneb, Tsion-Seguage, Sheha-Gomenege and Makesegnit Town in Gondar *Zuriya* District, northwest Ethiopia, from April to June 2017. Gondar Zuriya District is composed of hilly and plain landscapes, and the altitude ranges from 1750 to 2600 m above sea level. The study area has two malaria transmission seasons, which are the major transmission season (September to December) and the minor transmission season (April to May). In the study area, numerous individuals travel to malarious lowlands for temporary work, mainly during the rainy and spring seasons. A trend analysis (2002–2011) conducted in a neighboring district reported 23,473 malaria cases (75% *Plasmodium falciparum* and 25% *P. vivax*) with a fluctuating trend. From 2010 to 2011, *P. falciparum* prevalence decreased while *P. vivax* prevalence was increasing, indicating a trend shift [24]. Another study conducted in three districts, including Gondar *Zuriya* District, reported 61.3% malaria prevalence microscopically [25]. A recent spatio-temporal analysis in northwest Ethiopian districts revealed a purely temporal high malaria cluster from July 1, 2015 to December 31, 2016 and significant spatio-temporal malaria cluster were identified in Dembia District from January 1, 2014 to December 31, 2017 [26].

The characteristics of the study area, settings, and residential housing are described in detail elsewhere [22]. All the *kebeles* are within 12 km of Lake Tana, the largest lake in Ethiopia, bordering them in the southwest direction. The lake is the source of the Blue Nile and geo-located in 10.95°-12.78°N latitude and 36.89°-38.25°E longitude. The climate of Lake Tana basin is warm-temperate dominated by the tropical highland monsoon. Most rainfall (70–90%) falls between June and September. The water of Lake Tana is decreasing in volume and becoming increasingly turbid, resulting in the spread of water hyacinth. Houses in the study villages are predominantly of the mud-stick type with corrugated iron roofs. Sleeping mats made of hides are used in most rural HHs. Culicine mosquitoes, houseflies, fleas, bedbugs, cockroaches, crawlers, and spiders are the most common nuisance causing arthropods in local houses. Many of the villagers are subsistence farmers. Women are involved mainly in agriculture and domestic work.

### Study design

The present study employed a phenomenological qualitative methodology to explore the local contexts and factors that influence persistent use of LLINs. Phenomenology is a best approach and method of inquiry which helps to exhaustively describe, interpret, illuminate and qualify a phenomenon in everyday human life experiences [27, 28]. This methodology was chosen to explore the behavioral, demographic, socio-cultural, economic, livelihood, and housing situation, as well as net delivery related experiences of the community. Since LLINs utilization reveals lived experience of the communities, interpretive phenomenological approach was used. Descriptions of the participants’ LLIN utilization experiences were summarized and analyzed thematically.

### Sampling of study population

The study sites were purposefully selected to capture the views and practices of residents living in areas identified by the investigators facing challenges in LLINs utilization. The sample consisted of two groups: 23 community residents and 38 key informants. The sample size was estimated on the basis of richness, detailed nature and volume of data collected; relevant published literature; scope of study; and the amount of useful information obtained from each participant [29]. Saturation, the most widely used principle for qualitative sample size determination, was achieved with this large sample due to the heterogeneity of the participants and the broad scope of the study [30]. Proportional number of households were included from the 7 *kebeles.* Then, community residents comprised of household heads from a range of socioeconomic backgrounds and ages, and both genders and lactating women were sampled. They were interviewed about their LLINs utilization experiences and practices. Most of the study households (15 interviewees) owned LLINs and some others (8 interviewees) did not during the study period. The key informants were people with knowledge of malaria control and distribution of LLINs in the study communities and who knew the living conditions, beliefs, and behaviors. They were 5 health professionals responsible for the prevention and control of malaria at Makesegnit and Lemba health centers and Gondar Zuriya District Health Office, 8 health extension workers (HEWs) who worked in the 7 selected *kebeles*, 19 district/*kebele*/village leaders appointed by the local government, and 6 students. Village leaders were enlisted to recruit participants with support from a researcher to ensure a representative sample.

### Data collection procedures

Interviews were conducted in the local language, Amharic, and lasted up to 1 h each. The interview guide focused on reasons for not using LLINs persistently, inequitable use of LLINs among family members, compatibility of nets with sleeping spaces, sleeping time under an LLIN, perceived benefits and inadequacies of LLINs, and proper utilization of LLINs. The interviewers were trained in the use of the guide, asking open-ended questions, and probing for more detail. Questions were asked consistently during all interviews. All interviews were recorded on a digital voice recorder.

### Data analysis

Digital recordings of interviews were transcribed verbatim and translated from Amharic to English by the first author and local translators. Data management and analysis were done using NVivo Version 10 (QSR International, Melbourne, Australia). Thematic analysis is a flexible approach to identifying, analyzing, and reporting patterns of data and is compatible with both essentialist (realist) and constructionist paradigms [31]. The data were content-coded for thematic analysis. Initial coding was based on preset categories developed from the literature [13, 14] and emerging themes were derived from the data. The coded data were analyzed by querying to determine the frequency of occurring concepts, themes and relationships. Themes summarize the responses, and the words of the interviewees were presented in Word Clouds to illustrate key findings; quotes were taken from the interviews to illustrate the contextual quality of the findings. Textual and structural analyses were carried out to get comprehensive meanings of the lived experiences of the interviewees on LLINs utilization [32]. Textual analysis refers to the description of what is expressed by the interviewees while structural analysis stands for the interpretation of how it is expressed by the interviewees. Interpretive phenomenological analysis, which was used to understand lived experience of the interviewees about non-persistent utilization of LLINs and with how participants themselves make sense of their experiences, was employed [33].

## Results

### Socio-demographic description and **s**leeping time under LLINs

Of the 61 interviewees, the majority were males, adults, uneducated, and local community members (Table 1). Most informants reported usually going to sleep between 9 PM and 10 PM. One man said, *“After all the domestic animals get in their corrals, around 2100 h.”* Some individuals thought mosquitoes do not bite in the early evening.

**Table 1:** Demographic Characteristics of Interviewees

Characteristic	Frequency *n* (%)
Sex	
Male	33 (54.1)
Female	28 (45.9)
Age (years)	
15–25	9 (14.8)
26–45	35 (57.4)
> 45	17 (27.8)
Education level	
Uneducated (unable to read and write)	24 (39.3)
Primary	17 (27.9)
Secondary	6 (9.8)
College/university	14 (23.0)
Occupation	
Local leader	19 (31.2)
Student	6 (9.8)
Health professional^a^	5 (8.2)
HEW	8 (13.1)
Local community member^b^	23 (37.7)

^a^Malaria focal persons, Environmental Health Officer and District Malaria officers; ^b^Farmers and town residents

### Perceived benefits and shortcomings of LLINs reported by informants

Informants’ perceptions of the benefits and weaknesses of LLINs varied considerably. First, they evaluated them by their ability to kill arthropods such as houseflies, fleas, bedbugs, cockroaches, and spiders. Most informants commented that the currently distributed LLINs had not killed these arthropods although almost all acknowledged they had done so when new and up to 3 months old. Many informants attributed the decrease in killing ability of LLINs after several months to inadequate impregnation. Loss of killing ability was one of the reasons residents considered them ineffective and discontinued their use.

*Nets distributed previously had adequate insecticide. The current nets are inadequately impregnated. I think that they cannot serve for 3 years. They do not serve even for 2 years.* (Female, 38 years old, HEW in Sheha-Gomenege *Kebele*)

*The insecticide content of the current net is not a killer. It did not kill any arthropods. The current nets did not kill even mosquitoes that caused nuisance.* (Male, 42 years old, local leader in Sheha-Gomenge *Kebele*)*.*

Secondly, respondents rated LLINs based on their physical integrity. They reported that the nets quickly became dirty and faded; they were worn out due to repeated washing and broke after 6 months of use. Loss of physical integrity, especially breakage, appeared to be due to the impoverished living conditions in rural houses.

*The strength of LLIN is also not as such strong compared with the previous ones. It did not serve even for 2 years and they easily tear off in most HHs.* (Female, 35 years old, HEW in Tsion-Seguaj *Kebele*)

On the other hand, some informants reported that LLINs are very strong, adequate in size, and can serve throughout the stated lifetime if handled with care. They described the current LLINs as relatively smooth in texture and comfortable to the skin. However, a larger number of informants questioned the stated service length and the adequacy of the nets’ size; they considered them serviceable for 2 years at most if handled with care. Some stated that the large mesh size allowed mosquitoes to go through and complained that the nets were too small for large beds. Additionally, new nets were said to cause skin irritations when in contact with skin.

Regarding the mounting and shape of LLINs, many informants reported that they or others in their HHs who used them preferred the circular shaped nets because they were compatible with any sleeping space. Circular nets mounted at one point in the ceiling were simple to install and operate and easy to roll up during daytime and suspend at night. An environmental health officer and ex-malaria worker noted that “*circular shaped nets are durable and fit for thatched roof houses.*” Rectangular shaped nets, by contrast, were viewed unfavorably due to the difficulty of installing them and the large space they required. Thus, informants revealed that only HHs with beds in *chiqqa bet* (mud-stick houses) can comfortably hang and use the rectangular nets. However, rectangular nets were not preferred because their installation made holes and cracks in the mud walls.

*Even though the rectangular type [of nets] is suitable to use on beds, circular types (too long) are very comfortable to use on mats on the floor or on raised earthen platforms*. (Female, 35 years old, HEW in Layeye-Dugie *Kebele*)

*We have seen nets which are circular in shape in towns and this type is very good for rural residents too. It is tied only on one rope and easy to suspend and roll. It is very good.* (Male, 50 years old, main administrator of Sheha-Gomenge *Kebele*)

### Barriers to the persistent utilization of LLINs

**Fig. 1:**
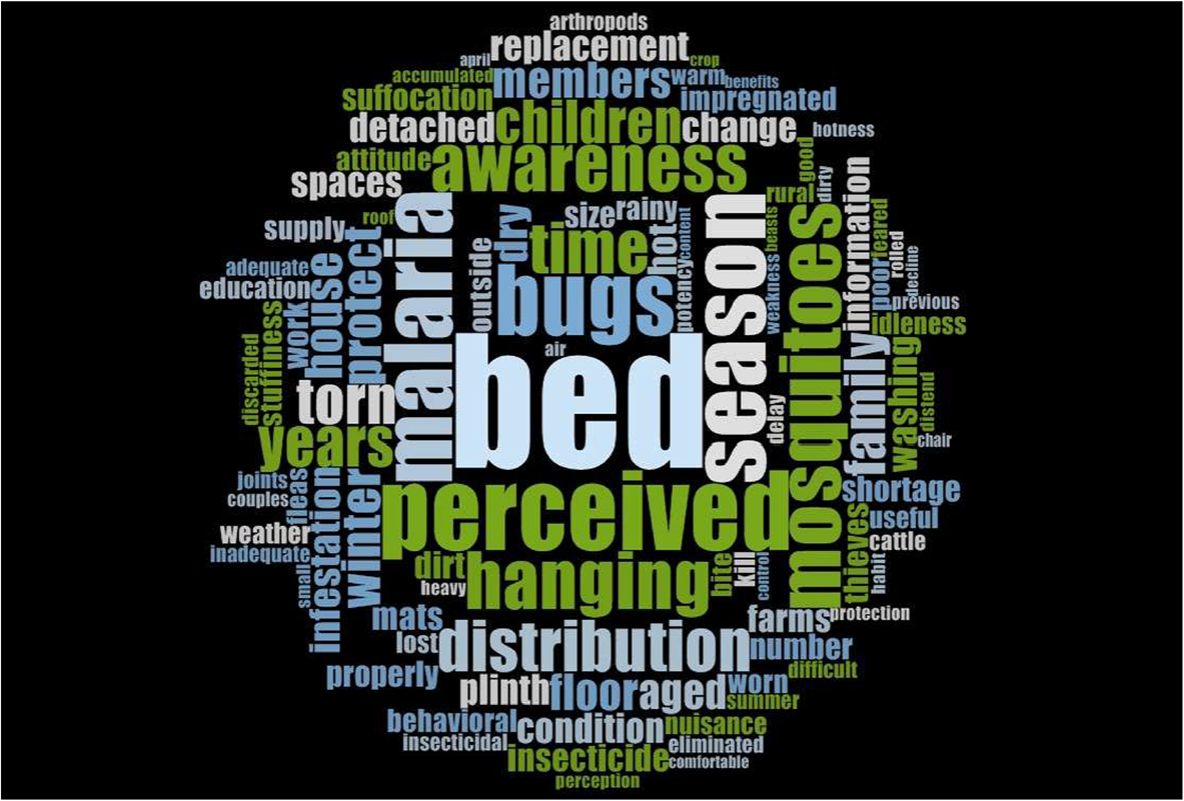
Word Cloud: 100 most occurring words, showing the phrase “bed bugs” as the most frequent one (NVivo Version 10)

During almost all interviews, bedbug infestation was mentioned and observed as a major deterrent for persistent use of LLINs (Fig. 1). The hanging corners of LLINs and the top inside of the nets harbored bedbugs. Additionally, bedbug urine and faeces were associated with soiling and pigmentation of LLINs. Bedbugs reportedly infested almost all houses in the study communities.

Perception of the ineffectiveness of LLINs in killing bedbugs and other arthropods was cited as a major barrier to persistent use of the nets. Some interviewees questioned the efficacy of LLINs that cannot protect against bedbugs and fleas beyond the first few months of use:

*LLINs killed bedbugs when they were new. When the net stopped killing bedbugs, we suspected that it also did not kill mosquitoes.* (Female, 55 years old, local leader in Makesegnit Town)

Community residents also indicated problems in receiving the nets as constraints for LLIN utilization. They cited insufficient supply of LLINs delivered to the HH, inequitable and late delivery, and irregular replacement of old nets. However, HEWs and some other informants argued that HHs received LLINs based on their family size per national guidelines. Informants also reported that all the HH members did not sleep under an LLIN because HH members did not follow the recommended guideline of sleeping in groups. Some HHs were denied LLINs because they did not meet the requirement of constructing a latrine.

Another barrier to persistent utilization of LLINs was the lack of adequate information about their proper use. Many residents reported having been informed that LLINs had to be impregnated every 6 months like conventionally treated nets. Others thought that simply hanging a net without adjusting it or using it as a bed sheet would protect from mosquito bites. Some residents did not use the nets after washing them due to the perception that they had lost their insecticidal ability during laundering.

In some houses, bed structure was a critical barrier to net utilization. Structures such as mud-made floors and sleeping platforms, chairs, verandas, and straw beds were unfit for rectangular nets. Rectangular net mounting was reported and observed to be impossible in short and narrow thatched-roof houses that are common in the study area.

A number of other barriers were also identified. Residents disliked the nets; they were stuffy, had an odor, caused allergic reactions, and made sleeping spaces dark. Elderly residents often forgot to suspend them and busy farmers felt they could not take time from their work to hang them. LLINs were inconvenient for residents who traveled and those who were forced to sleep outside to protect their cattle and other resources at night.

Some informants reported that residents believed mosquitoes and the threat of malaria had been reduced or eliminated, and they associated this belief with a decline in the use of LLINs. The common perceptions that mosquitoes are absent during the dry season and that malaria is a disease of the past impede LLIN use despite efforts of the key informants to facilitate persistent utilization of LLINs.

### Unintended and perceived collateral uses of LLINs

Informants observed and reported unintended use of LLINs, either misuse or repurposed use. LLINs were used for unintended purposes when they were old, dirty, torn, or had otherwise lost their physical integrity and after washing. Unsafe use and poor management practices were reportedly the major reasons for widespread damage, leading to repurposed uses.

Repurposed uses of LLINs included covering of pumpkin and pepper seedlings, drying of cereals, protecting and assembling straw and cereal harvest residues, and bagging garlic for transport to markets on donkey carts. Torn nets were repurposed for fencing and made into ropes for binding and tethering purposes.

**Fig. 2:**
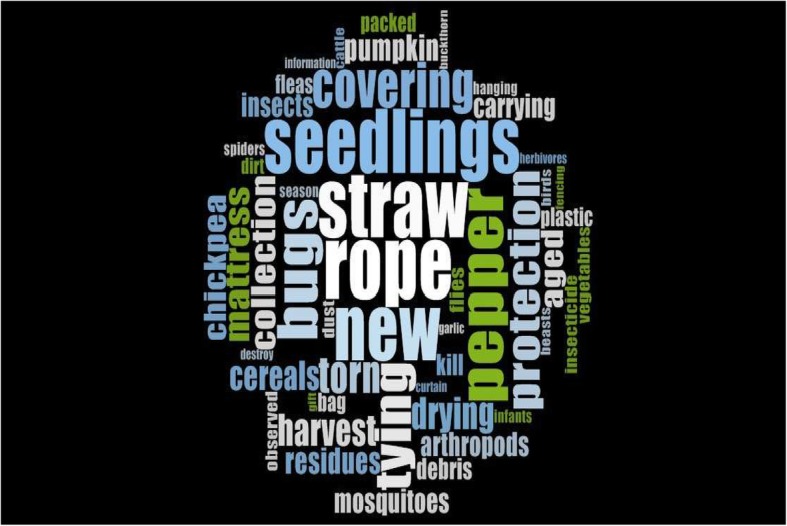
Word Cloud: 50 most occurring words showing terms like “rope”, “seedlings” and “straw” (NVivo Version 10)

**Fig. 3:**
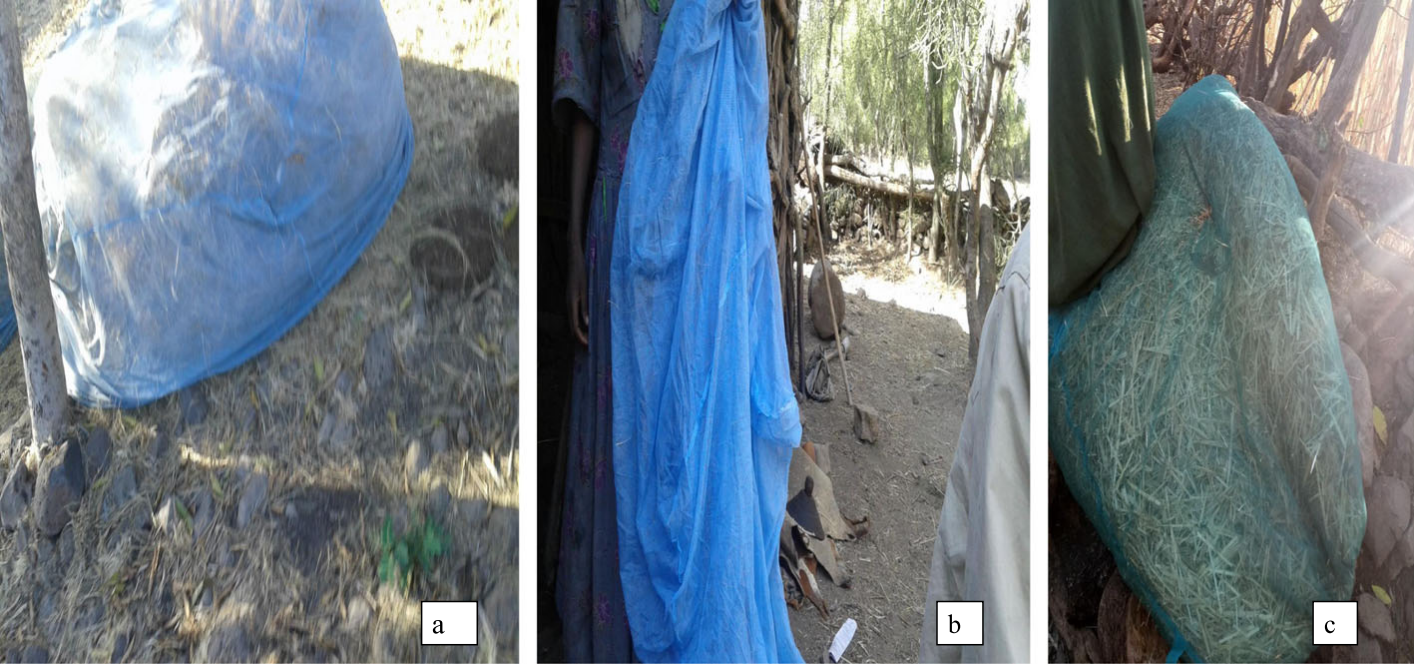
Misuse of new LLINs: **a**) Used for protection of tef (*Eragrostis tef*) straw from herbivore domestic animals. **b**) Put as packed in its plastic bag. **c**) Used as a sleeping foam by filling with straw

The key informants as well as community residents reported misusing LLINs. Residents used new LLINs for domestic needs, such as for protecting and bagging straw and cereal harvest residues; protecting pepper and eucalyptus seedlings; and drying cereals, buckthorn *(gesho*), malts (*gebs)* and pepper fruits. Participants placed LLINs under mattresses and as bed sheets to protect them from bedbugs and used them to cover latrine walls and make ropes (Figs. 2 and 3). In rare cases, the nets were used as head scarves, curtains for windows, and doors. Some residents used one net for mosquito protection and the remaining ones for unintended purposes. Misuse of nets also included giving them to friends or relatives as gifts, placing them in boiling water to kill bedbugs, and storing them in plastic bags (Fig. 3). Some informants reasoned that because malaria had been eliminated, using nets for other purposes made sense. However, several informants did not use new LLINs for unintended purposes and advised residents to also use them for their proper purpose.

Both key informants and residents saw the insect control ability of LLINs as a collateral benefit beyond protection from mosquito bites and malaria. LLINs can effectively destroy insects and other arthropods when new. There was a perception that LLINs must protect primarily against bedbugs and fleas, and additionally against other arthropods. Residents with infants reportedly used the nets day and night for protection against house flies, crawlers, debris, and dust. Cessation of insecticidal activity of LLINs against bedbugs after 2 or 3 months was the most frequent and serious complaint of community informants. Control of bedbugs was the commonly perceived primary collateral use of LLINs:

*Instead of hanging the net, they used it under the mattress since they believed the insecticide on the net killed bedbugs. They primarily used to protect from bedbugs despite they knew it protects from mosquitoes.* (Malaria focal person in Lemba HC)

### Informants’ suggestions to increase LLIN use

**Fig. 4:**
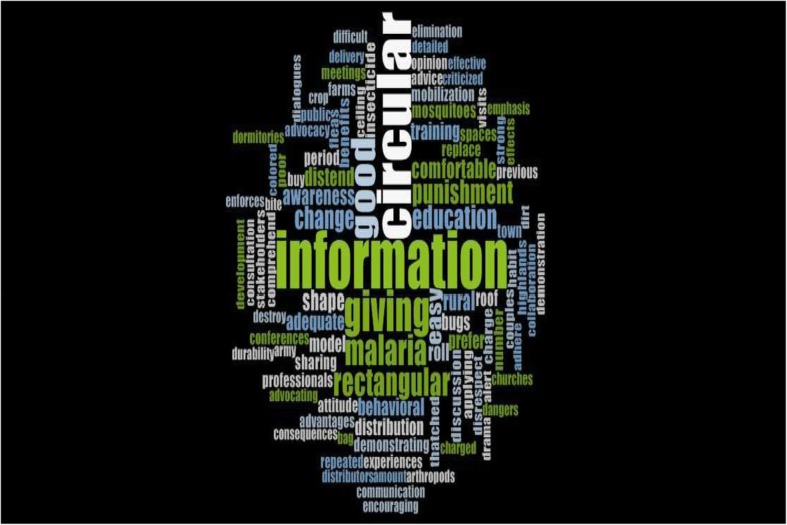
Word Cloud: 100 most occurring words showing the term “circular” as more frequent than “rectangular” (NVivo Version 10)

Informants and the wider community in the study villages had color preferences for bed nets. In addition, participants indicated a strong preference for circular nets over rectangular ones as the circular nets are easier to install and use (Fig. 4). Circular types were frequently suggested for rural residents because their sleeping spaces are not uniform or comfortable, primarily because of their low socio-economic status, social and cultural issues, and seasonal changes. Some informants proposed a portable net that can be used in any space, similar to a sleeping bag, because individuals may sleep outside or away from home.

Suggestion by informants indicated that information, education and communication (IEC) and behavior change communication (BCC) interventions should always go along with distribution of LLINs and during the active life span of nets in communities’ utilization. Informants recommended improving the effectiveness of LLINs by replacing them every year or 2 years, re-impregnating them every 6 months, or increasing the amount of insecticide used. Prolonging the ability of LLINs to kill fleas and bedbugs was said to increase persistent use.

Some informants proposed reinforcing persistent use by establishing rules for LLIN use and imposing punishment or awarding incentives for following the rules. Other informants argued that the introduction of punishment would create a social crisis for HEWs and decrease net use. Inter-sectoral networking was suggested as a tool for applying positive punishment.

### Follow-up, supervision and evaluation of LLINs utilization

On one occasion, the Malaria Prevention and Control District Office temporarily enforced punishment for households that did not use nets or utilized them for unintended purposes. Generally, however, strict follow-up, supervision, and evaluation of the use and efficacy of LLINs have reportedly not been carried out in the study area:

*HEWs simply alerted us to take the nets. They simply told us to use them properly. There is no regular follow-up and supervision on how the community utilizes the net.”* (Male, 40 years old, community member of Dugie Village, Layeye-Dugie *Kebele*)

The lack of follow-up and supervision constitutes a critical gap in malaria control efforts. The absence of follow-up was attributed mainly to recent reduction in malaria occurrence. Another reason for lack of follow-up was poor community acceptance of local mobilizers delivering information about net utilization. Inadequate HEW personnel was reported as a reason for the weakness of malaria interventions in the community. Informants from the study community reported that implementing comprehensive interventions is beyond the capacity of HEWs because they are ignored by local officials. Nevertheless, some community residents praised the efforts of HEWs to obtain persistent utilization even in the absence of behavioral change.

Some informants preferred bi-annual indoor residual spraying (IRS) over using LLINs. They reasoned that IRS was better for people with asthma, elderly people, and those unable to hang nets; IRS had benefits in homes with faulty plastering and in newly constructed houses. Some informants preferred combining IRS and LLINs.

## Discussion

The findings of this study are organized into four themes: (1) perceived benefits and shortcomings of LLINs, (2) unintended and perceived collateral uses of LLINs, (3) factors that influence persistent utilization of LLINs, and (4) informants’ suggestions for increasing LLIN use.

Study participants gave high marks not for malarial protection benefits of bed nets, but for their collateral benefit of protecting against arthropods such as house flies, crawlers, fleas, bedbugs, cockroaches, and spiders in the first 3 months of use. Similar information was reported from elsewhere in Ethiopia [14] and Uganda [13]. Loss of insecticidal effectiveness of LLINs after several months, considered a critical limitation, was reported elsewhere in Ethiopia [4, 14] and Kenya [9], where users were not aware of the chemical impregnation of nets and reported them ineffective as physical barriers. In our study, informants noted that LLINs quickly became dirty, faded, worn out due to repeated washing, degraded under the conditions of rural houses and mice infestation, and were no longer functional after 6 months. Doda and colleagues [14] also reported LLINs non-functional and repurposed after 6 months. Solomon et al. [21] reported that LLINs had a 12-month median functional survival time and that successive laundering significantly increased feeding success and survival rates of *Anopheles gambiae* sensu lato [34]. However, WHO indicates the serviceable life span of LLINs to be 3 years [19, 20]. Our finding that users reported that exposure to the chemicals caused skin irritation was also reported in several others studies [4, 18]. Regardless of study population characteristics, some key informants and most community residents questioned the strength and size, including the mesh size of the current LLINs in this study. Other studies also reported small net size, weak physical integrity, and big mesh size as shortcomings of LLINs [11, 15, 35]. However, LLINs were highly credited for their strong texture, wide square shape and large mesh size in other studies [15, 35].

In this study, key informants and residents preferred circular-shaped nets due to their adaptability to any sleeping space and ease of operation; this preference was also reported by other studies [14]. The challenges and shortcomings of rectangular nets—the complexity of installation, the need for a wide area, and usefulness only with beds in mud houses (although their installation damages the mud walls)—were also reported by other studies [4, 13, 14].

Our findings verified that the most common repurposed uses of nets were connected to the prevailing local livelihood and socio-economic conditions. These uses peaked during the harvest season. Other studies documented similar repurposed uses of nets [14, 36, 37]. In our study, residents used LLINs for other purposes when they determined the nets had ceased to serve their intended purpose due to age or loss of mosquito-killing capacity; this was also reported in other studies [14, 38]. Repurposing LLINs is an increasing phenomenon in villages around Lake Tana. Similar studies elsewhere in Ethiopia and Africa reported that residents repurpose nets for various uses [4, 6, 9, 14, 38, 39].

We found misuse of nets, even when new, for a wide range of purposes. Similar misuse was practiced in other communities in Ethiopia and elsewhere in Africa [4, 14, 40]. The reasons cited for the misuse of LLINs in our study were the perception that malaria has been controlled and the inability to hang nets in all sleeping spaces. Similar perceptions that led to misuse of nets were reported in other studies [14].

In our study, the main collateral uses of LLINs were for killing and warding off of arthropods, protection from hens, and protection from dust and debris, primarily for infants. Such uses, particularly fighting bedbug infestation, motivated residents to use nets persistently for the first few months. Similarly, Birhanu et al. [4], Strachan et al. [13] and Doda et al. [14] reported that community residents obtained diverse collateral benefits from LLINs beyond the prevention of malaria and the nuisance of mosquitoes. In this context, promoting persistent LLIN use can be most effective if accompanied by interventions that can eliminate bedbug infestation. WHO reported that bedbugs contribute indirectly to the ineffectiveness of malaria interventions and recommended the use of insecticides such as pyrethroids, diazinon, bendiocarb, and dichlorvos [41]. However, bedbugs have already developed resistance to these insecticides and studies recommended consideration of other disinfestation mechanisms [42].

Our study found that inadequacy of nets in a HH, inequitable distribution, and absence of on-time replacement were first-line barriers to LLIN utilization. Such delivery-related constraints were reported by similar studies in Ethiopia [4] and Zanzibar [17]. The finding that infants and pregnant women were most likely to use LLINs persistently was also seen in other studies [4, 14, 43].

Persistent utilization of LLINs by villagers around Lake Tana was to a great extent determined by socio-demographic and socio-cultural characteristics, behavioral factors and misconceptions, weather conditions, and livelihood and economic factors. Equivalent factors were reported in other communities [4, 11, 13, 14, 36, 44–49]. The perception that malaria is no longer a problem contributed to non-persistent net use was also mentioned in other studies [4, 14, 18]. Our finding that dirtiness, aging, and deterioration of LLINs, together with the perception that LLINs lose their insecticidal activity after washing, corroborates findings of other studies [4, 14]. In addition, failure of the nets to protect against bedbug and flea infestations after a few months was a predominant barrier of persistent use of bed nets in the study communities. This was also cited as a serious problem in other studies elsewhere in Ethiopia [14], Uganda [13], and Rwanda [39].

Residents’ limited information about LLINs, malaria transmission, and vectors resulted in misperceptions and negligence, which in turn led to non-adherence to net use. Other studies confirmed such analytical associations [14, 49–52]. Berhanu et al. [4] and Doda et al. [14] similarly reported a behavioral shift away from LLIN use due to perception of low malaria risk, saving of nets for future use; lack of awareness and negligence accounted for non-consistent use of LLINs. In addition, lack of clear information about the life span of LLINs constrained bed net use, corroborating the findings of other studies [4, 9, 14, 53]. Misperceptions about the effectiveness of insecticides after repeated washing were major barriers to persistent use. These misconceptions were reported from other communities in Ethiopia [4, 14], Ghana [54] and Kenya [9]. A study in south-central Ethiopia showed that LLINs were effective up to 24 months [21].

Sleeping spaces without beds and incompatible bed structures were associated with the failure to use nets persistently. Even small, thatched-roof houses were observed and reported to be incompatible with use of rectangular-shaped nets. Similar challenges were identified in other studies [4, 13, 36]. These sleeping arrangements are an indication of poverty.

Informants in our study suggested greater persistent utilization of LLINs may be achieved through wider distribution of circular or conical nets instead of rectangular nets, a suggestion also reported in studies elsewhere in Ethiopia [55] and Uganda [39]. Informants also proposed provision of a portable net, such as a self-supporting and sealed pop-up net, that can be used in any space indoors or outdoors. Such a net might be easier for older children and women to assemble and may also prevent, for example, soldier ants and reptile infestation. Other studies also recommended the development of portable, user friendly nets that do not need external supporting structures [11, 56]. Another way to increase persistent use of mosquito nets, resolving technical difficulties related to mounting and using a net, may best be addressed at the manufacturer level.

This analysis of 61 interviews with community residents and key informants offers new insights into perceived benefits and shortcomings of LLINs, unintended and collateral uses of LLINs, and reasons for non-persistent use, including bedbug infestation, wearing out of LLINs, and misconceptions about LLINs. These insights may help Gondar Zuriya District Health Office take measures to optimize persistent LLIN use to support malaria elimination efforts.

## Conclusion

We conclude that wrongly perceived weaknesses of LLINs were common; rectangular LLINs were considered inferior to conical nets; unintended uses were widespread; collateral benefits of nets were considered important; and behavioral, socio-cultural, livelihood, housing, and net delivery-related factors generally impeded persistent use of LLINs. Suggestions made by informants for increasing persistent LLIN use, including the provision of nets that are structurally compatible with sleeping spaces and the delivery of adequate information about malaria vectors and the properties and proper uses of LLINs to the community indicate substantial accumulation of knowledge that may guide effective LLIN programs in the study area.

## Data Availability

Data and all the materials will be available from the corresponding author upon request.

## Abbreviations

BCC: Behavior change communication
HEWs: Health extension workers
HHs: Households
IEC: Information, education and communication
IRS: Indoor residual spraying
LLINs: Long-lasting insecticidal nets
